# MYB Transcription Factor *OsC1^PLSr^* Involves the Regulation of Purple Leaf Sheath in Rice

**DOI:** 10.3390/ijms24076655

**Published:** 2023-04-03

**Authors:** Ting Zou, Xinyi Wang, Tong Sun, Huazhen Rong, Linxuan Wu, Jing Deng, Tao Guo, Hui Wang, Jiafeng Wang, Ming Huang

**Affiliations:** Guangdong Provincial Key Laboratory of Plant Molecular Breeding, South China Agricultural University, Guangzhou 510642, China

**Keywords:** rice, purple leaf sheath, *PLSr*, transcriptional activity, CRISPR/Cas9

## Abstract

Although several regulators associated with purple traits in rice have been identified, the genetic basis of the purple sheath remains unclear. In the present study, F2-1 and F2-2 populations were constructed using purple sheath (H93S) and green sheath (R1173 and YHSM), respectively. In order to identify QTL loci in purple sheaths, BSA analyses were performed on the two F2 populations. A crucial QTL for purple sheath was identified, tentatively named qPLSr6, and was located in the 4.61 Mb to 6.03 Mb region of chromosome 6. Combined with expression pattern analysis of candidate genes, *LOC_Os06g10350* (*OsC1^PLSr^*) was suggested as a candidate gene. The homozygous mutant KO-1 and KO-2 created through CRISPR/Cas9 editing, lost their purple leaf sheath. The RT-PCR revealed that *OsC1^PLSr^*, anthocyanin synthase (ANS), diflavonol-4-reductase (DFR), flavanone-3-hydroxylase (F3H), and flavanone-3′-hydroxylase (F3′H) expression levels were dramatically down-regulated in the mutants. The yeast report system indicated that the 145–272 aa region at the C-terminal of OsC1^PLSr^ is a positive transcriptional activation domain. The results indicated that OsC1^PLSr^ synthesized anthocyanins by regulating the expression of ANS, DFR, F3H, and F3′H. This study provides new insights into the genetic basis of the purple sheath.

## 1. Introduction

The general kinds of anthocyanin are geranium pigment, cornflower pigment, anthocyanin, morning glory pigment, mallow pigment, and paeoniflorin, and they are unevenly distributed in plants [1,2]. Anthocyanins belong to secondary metabolites, which stability is affected by the self-chemical structure and environmental factors. Structural genes mainly encode crucial enzymes in the anthocyanin biosynthesis pathway, such as phenylalanine ammonialyase (PAL) [3], cinnamic acid hydroxylase [4], chalcone synthase (CHS) [5], chalcone isomerase (CHI) [6], diflavonol-4-reductase (DFR) [7], anthocyanin synthase (ANS) [8]. Additionally, regulatory genes mainly encode transcription factors to regulate the spatio-temporal expression of structural genes in the anthocyanin biosynthesis pathway. At present, the critical transcription factors, including MYB, bHLH, and WD40 proteins, form a complex to modulate anthocyanin synthesis gene transcription for plant cell pigmentation [9,10,11].

The anthocyanin accumulation induced the purple trait in distinct rice organs. *OsPL6* locates on the short arm of chromosome 6, and upregulate-expression of *OsPL6* led to purple accumulation in the leaf [12]. *OsB2* is related to regulating anthocyanin accumulation. Ectopic expression of *OsB2* generates the production of purple or black pericarp in rice [13]. The genome-wide association study and transcriptome analysis showed that *OsC1, OsRb,* and *DFR* were identified as the determinants of anthocyanin biosynthesis in rice leaves [14]. Through bulked segregant analysis with next-generation sequencing (BSA-Seq) and transcriptome sequencing (RNA-Seq) strategies, identifying a recessive gene *plr4*, which was regulating purple leaf in rice and was located near the 27.9–31.1 Mb interval of chromosome 4 [15]. The *C-S-A* gene system controls hull pigmentation in rice. *C1* is a color-producing gene that produces brown when acted alone but purple when combined with *A1*. In addition, *S1* interacts with *C1* to activate the expression of the gene *A1*, then producing purple and brown hulls [16].

Despite multiple regulators responsible for the purple trait have been characterized, molecular detail of purple leaf sheath genes remains unclear. Previous research reports that the purple leaf sheath of rice was controlled by one or two pairs of genes through genetic analysis. Recently, researchers found *Ra*/*Rb* and *OsC1*, located on chromosomes 1 and 6 [17,18], which regulate anthocyanin accumulation in rice leaf sheaths. Through yeast two-hybrid analysis, *OsC1* was shown to interact with Rb1/Rb2 [17]. Although *OsC1* was mapped to produce rice purple leaf sheath, there has been little functional analysis of *OsC1* due to a lack of genetic transformation validation.

Therefore, this study performed BSA-Seq analysis using two F2 populations; the *qPLSr6* QTL was identified, and the candidate gene encoding MYB protein (OsC1^PLSr^) was functionally validated. Knockout homozygous mutants had a purple leaf sheath loss phenotype. We found that the OsC1^PLSr^ regulates anthocyanin synthesis through structural genes *ANS*, *DFR*, *F3H*, and *F3′H*. The results provided a theoretical basis for revealing the regulatory role of *OsC1* in the purple leaf sheath.

## 2. Results

### 2.1. Observation of Leaf Sheath Phenotype

**Figure 1:**
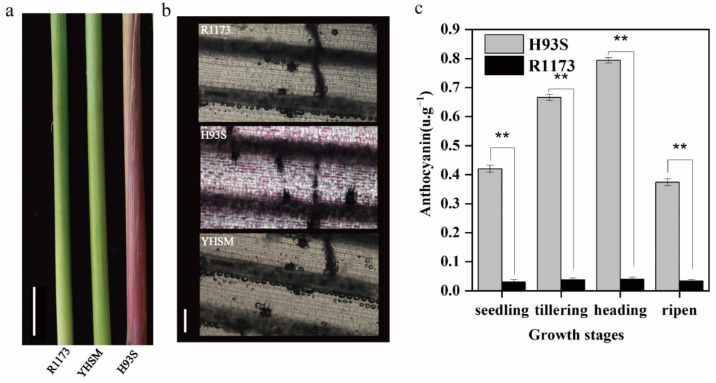
Phenotypes and total anthocyanin contents of purple and green leaf sheath in rice. (**a**), Leaf-sheath phenotypes of parental lines, the scale bar is 7.5 cm. (**b**), The leaf sheath longitudinal sections of parental lines, the scale bar is 100 μm. (**c**), Total anthocyanin contents in leaf sheath of H93S and R1173 at all growth periods. Values shown are means ± SD, ** indicates a significant difference according to the *t*-test (*p* < 0.01) between H93S and R1173.

According to phenotypic analysis, H93S, R1173, and YHSM have purple, green, and green leaf sheaths, respectively (Figure 1a). Observation with a digital microscope showed that a large number of anthocyanins were distributed in the leaf sheaths of H93S, while the distribution of anthocyanins was rarely screened in R1173 and YHSM (Figure 1b). Consistently, anthocyanin accumulation in the leaf sheaths of H93S was significantly higher than R1173 (Figure 1c). In the two F2 segregating populations, the color of the leaf sheath could be categorized into purple and green, and the expected Mendelian segregation ratios were both 3:1 (Table 1). These results indicated that the anthocyanin content is positively correlated with the leaf sheath color, implying a complete dominant gene controls the purple leaf sheath trait.

### 2.2. Identification of Candidate Regions for Controlling Leaf Sheath Color by BSA-Seq

A total of 60.72 + 60.42 Gbp of raw data were obtained and filtered into 58.47 Gbp and 57.67 Gbp of clean data from the F2-1, and F2-2 populations, respectively. Through the analysis of the data quantity and quality of seven samples (two PLSBs, two GLSBs, and three parents), it found that Q30s were all above 90.00%, and the GC content of the seven samples’ clean data ranged from 44.33% to 44.77% (Table 2). The average reading depth of seven samples ranged from 25× to 40×. According to sequencing data, 97.96–98.58% of the sequences can be successfully aligned to the reference genome R498. These results illustrated that all samples were of good sequencing quality and could be used for subsequent variation analysis. After filtering low-quality SNPs and InDels, finally, 675,512 SNPs and 140,374 InDels with credible and high quality were obtained from the F2-1 population, 466,796 SNPs and 96,738 InDels with credible and high quality from the F2-2 population (Table 3).

**Figure 2:**
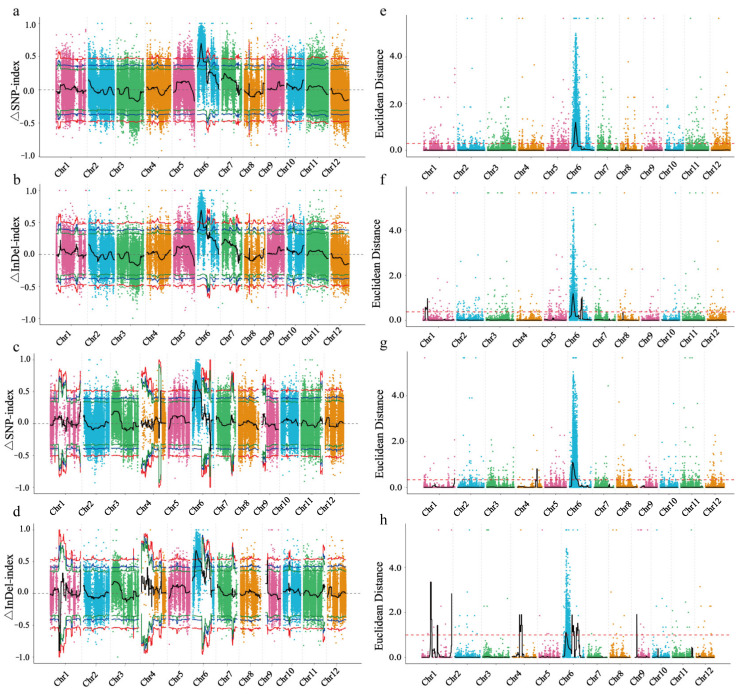
The ΔAll-index and ED are distributed on chromosomes of two populations. (**a**), Distribution of ΔSNP-index values of F2-1. (**b**), Distribution of ΔInDel-index values of F2-1. (**c**), Distribution of ΔSNP-index values of F2-2. (**d**), Distribution of ΔInDel-index values of F2-2. (**e**), Distribution of ED-associated values of F2-1 by SNPs. (**f**), Distribution of ED-associated values of F2-1 by InDels. (**g**), Distribution of ED-associated values of F2-2 by SNPs. (**h**), Distribution of ED-associated values of F2-2 by InDels. (**a**–**d**), The colored dots represent the calculated ΔAll-index value, and the black lines represent the fitted ΔAll-index value; the red lines represent the threshold line with a confidence of 0.99, the blue lines represent the threshold line with a confidence of 0.95, and the green lines represent the threshold line with confidence of 0.90. (**e**–**h**), The colored points represent the ED value of each SNP or InDel, the black lines represent the fitted ED value, and the red dashed lines represent the significant correlation threshold. The higher the ED value, the better the correlation effect of this point.

The ∆All-index (∆SNP-index and ∆Indel-index) and ED (Euclidean Distance) were calculated for variation analysis. The charts were plotted from the ∆All-index and ED (Figure 2). In the F2-1 population, the interval for the candidate genes exceeding the threshold value was identified on the 3.33–8.72 Mb of chromosome 6 covering 1415 genes by SNP association analysis, while it was identified on the 3.37–8.55 Mb of chromosome 6 covering 1274 genes by InDel association analysis (Figure 2a,b,e,f; Table 4). Thus, the interval of the F2-1 population was identified on the 3.37–8.55 Mb of chromosome 6 and had 1274 genes. In the F2-2 population, the interval for the candidate gene exceeding the threshold value was identified on the 4.03–9.06 Mb of chromosome 6 and had 1257 genes by the SNP association analysis, and on the 4.61–6.03 Mb of chromosome 6 and had 415 genes by the InDel association analysis (Figure 2c,d,g,h; Table 4). Thus, the interval of the F2-2 population was identified on the 4.61–6.03 Mb of chromosome 6 and had 415 genes. Both the populations were located on the 4.61–6.03 Mb of chromosome 6. These results suggested that the candidate genes of the purple leaf sheath of H93S were located in the 1.42 Mb region of chromosome 6 from 4.61 Mb to 6.03 Mb.

### 2.3. Analysis of Candidate Genes

**Figure 3:**
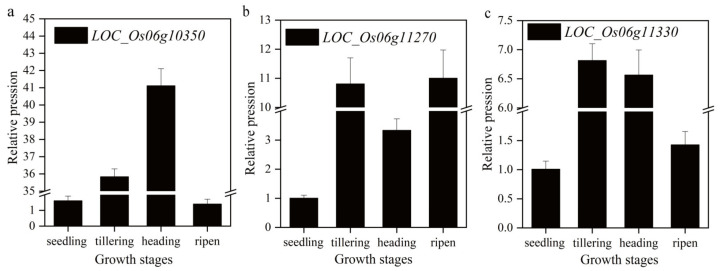
The transcript levels of the three candidate genes at the four growth stages of H93S. (**a**), *LOC_Os06g10350*. (**b**), *LOC_Os06g1035011270*. (**c**), *LOC_Os06g11330*.

There were 415 genes with the 1.42 Mb region of chromosome 6. Among the 415 genes, 27 were expressed in rice leaf sheath by the public date expression spectrum CREP analysis (Table 5). Then, annotated by Rice Genome Annotation Project, three genes (*LOC_Os06g10350*, *LOC_Os06g11270*, and *LOC_Os06g11330*) were involved in the anthocyanin synthesis pathway and were considered as candidate genes (Table 5). qRTPCR results showed that the expression pattern of *LOC_Os06g10350* in rice leaf sheath was “up-up-up-down” (Figure 3a) at four growth stages, which was consistent with the change of anthocyanin content in the four growth periods of H93S, and the expression level reached the peak at heading stage. While the expression patterns of *LOC_Os06g11270* and *LOC_Os06g11330* were “up-up-down-up” and “upup-down-down”, respectively (Figure 3b,c). SO, *LOC _Os06g10350* was considered to be a strong candidate for the *OsC1^PLSr^* locus, which is co-located with *OsC1* [18].

### 2.4. Knockout of OsC1^PLSr^ Results in No Purple Leaf Sheath Phenotype

**Figure 4:**
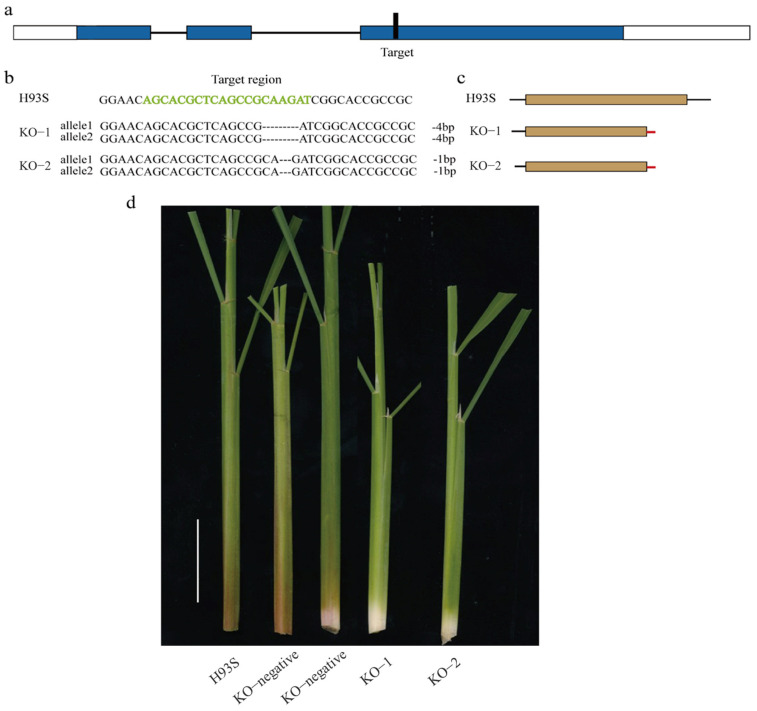
Knocking–out (KO) of *OsC1^PLSr^* in H93S by CRISPR/Cas9 genomic editing system. (**a**), The schematic representation of the target site position. (**b**), Sequencing analysis of target regions of mutant lines. (**c**), Amino acid changes in mutant lines. (**d**), The whole plant of H93S and the KO plants. The KO–negative failed to Knocking–out the *OsC1^PLSr^* gene in H93S by CRISPR/Cas9 genomic editing system. The scale bar is 5 cm.

**Figure 5:**
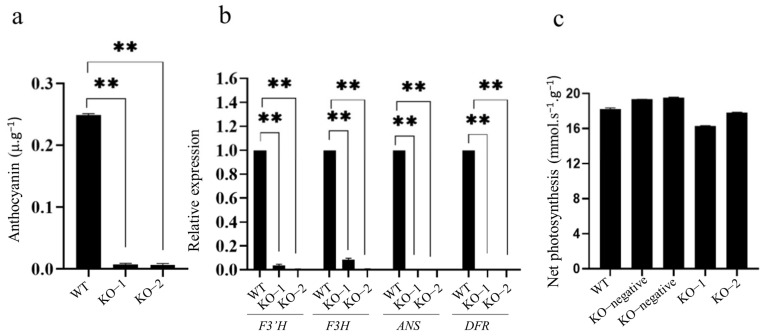
Analysis of transgenic plants. (**a**), The anthocyanin content of mutant lines and H93S. (**b**), shows the different expression levels of the genes *F3′H*, *F3H*, *ANS* and *DFR*, respectively. (**c**), The photosynthesis in mutant lines, H93S, and negative transgenic plants. Values shown are means ± SD, ** indicates a significant difference according to the *t*-test (*p* < 0.01) between WT and the KOs.

To verify the function of *OsC1^PLSr^* in H93S, we used the CRISPR/Cas9 genomic editing system to knockout *OsC1^PLSr^* in H93S to obtain homozygous lines KO-1 and KO-2 (Figure 4a). The KO-1 and KO-2 harbored allelic homozygous insertions or deletions of base pairs, which caused premature termination of the OsC1^PLSr^ protein (Figure 4b,c). Anthocyanin contents were measured in transgenic plants at tillering stage. The anthocyanin contents in the leaf sheath of mutant lines were significantly lower than H93S (Figure 5a). Besides, the relative expression of structural genes *DFR, ANS, F3H,* and *F3′H* in mutant lines was down-regulated (Figure 5b). Further phenotypic identification showed that the leaf sheath color was green of all these mutants (Figure 4d). In addition, the photosynthesis of mutant lines and H93S was not significantly different (Figure 5c). These results demonstrated that the two domains of *OsC1^PLSr^* play a critical role in inducing anthocyanin biosynthesis. In addition, the anthocyanin biosynthetic genes, *DFR, ANS, F3H,* and *F3′H,* were up−regulated by the activation of Myb transcription factor gene *OsC1^PLSr^*, guided to the accumulation of anthocyanin in H93S.

### 2.5. Structure Analysis, Transcription Activity Analysis, and Toxicity Detection

**Figure 6:**
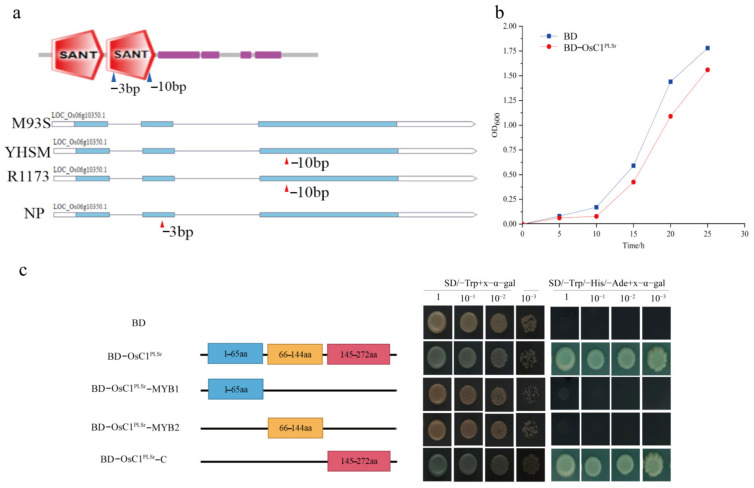
Structure analysis, transcriptional activation characteristics, and toxicity detection assay. (**a**), Structure deletion site analysis of *OsC1^PLSr^*. (**b**), Toxicity detection of OsC1^PLSr^ protein. (**c**), The full-length ORF of OsC1^PLSr^ and three truncated mutants (OsC1^PLSr^-MYB1, OsC1^PLSr^-MYB2, OsC1^PLSr^-C) were fused with pGBKT7, and the transformed AH109 yeasts were selected from SD/-Trp + x-α-gal medium and SD/-Trp/-His/-Ade + x-α-gal medium, respectively. The empty BD vector was used as a negative control. “SANT” means “SANT SWI3, ADA2, N-CoR, and TFIIIB’’ DNA-binding domains.

H93S with purple leaf sheath possessed a full-length DNA sequence of *OsC1^PLSr^* encoding 273 amino acids. Whereas R1173 and YHSM with green leaf sheath had a 10-bp deletion in exon 3 and only 207 amino acids. There was a 3-bp deletion in exon 2 in the green leaf sheath of Nipponbare (Figure 6a). *OsC1^PLSr^* contains two conserved domains (R2-MYB and R3-MYB), and the two deletion sites were located in the R3-MYB domain (Figure 6a). The results revealed that an aberrant R3 MYB domain of *OsC1^PLSr^* failed to induce anthocyanin synthesis in R1173, YHSM, and Nipponbare.

To further study the function of *OsC1^PLSr^,* by the toxicity detection assay, we found the growth curve of the yeast cells containing the negative control pGBKT7 (BD) was higher than pGBKT7-OsC1^PLSr^ (BD-OsC1^PLSr^). This result demonstrated that the protein encoded by *OsC1^PLSr^* had a slightly toxic effect on the growth of yeast cells (Figure 6b). In addition, we constructed various regions of the OsC1^PLSr^ protein by using the yeast report system. These regions included the MYB1 domain, MYB2 domain, C-terminal region, and the full-length OsC1^PLSr^. The regions were fused to the *GAL4* DNA-binding domain in the pGBKT7 vector, respectively. Then, they were all transformed into AH109 yeast cells. These results indicated that the amino acids 145–272 region of the C-terminal in *OsC1^PLSr^* were responsible for transcriptional activation activity; the MYB domain did not perform transcriptional activation function (Figure 6c).

## 3. Discussion

Anthocyanins are flavonoids, which are secondary metabolites produced by plants that generally increase with the stimulation of biotic or abiotic stress [19]. Target QTL can be located rapidly and precisely using BSA in conjunction with high-throughput genotyping technologies from next-generation sequencing. In this study, we first constructed two F2 populations and then used BSA-seq technology for preliminary mapping. Both F2 populations were used to map the purple leaf sheath gene. It is possible to swiftly and precisely identify the candidate interval by association analysis of the two populations. To accurately screen potential genes. There was no extensive population mapping done. As a result, labor is saved.

R2R3-MYB transcription factors have been clarified to participate in the determination of anthocyanin synthesis and regulation in plants. The gene *TT2* of Arabidopsis encoding R2R3-MYB protein regulates proanthocyanidin biosynthesis by activating the expression of *ANR*, while the closely related MYB PAP4 protein (AtMYB114) specifically activates *UFGT* expression and regulates the anthocyanin biosynthesis [20]. However, few studies have reported how MYB domains regulated the synthesis of anthocyanin in the leaf sheath of rice. In this study, we found that two MYB domains of OsC1^PLSr^ were essential for anthocyanin synthesis in the leaf sheath. We found that only the amino acids 145–272 region of the C-terminal in OsC1^PLSr^ were responsible for transcriptional activation activity, while the MYB domain has no transcriptional activation effect, which indicated that MYB domain was only responsible for directly binding DNA sequence with target gene, but not for its own transcriptional activation effect. Therefore, it is speculated that the R2R3-MYB transcription factor OsC1^PLSr^ in H93S may also bind to the promoters of *DFR*, *ANS*, *F3H,* and *F3′H* genes to activate the expression of these four structural genes. However, through the analysis of 2 Kb promoters upstream of *DFR* and *F3′H* genes by the Plant CARE of promoter prediction analysis website, the results showed that the promoters of *DFR* and *F3′H* genes contained light responsive MYB binding site (AACCTAA). Therefore, *OsC1^PLSr^* may directly regulate the expression of *DFR* and *F3′H*. The regulatory effect of *OsC1^PLSr^* on *DFR* and *F3′H* gene expression needs to be further studied.

In addition, the purple leaf sheath is the most stable, early, and visible trait for rice tissues, which is an excellent visual marker-trait [21]. There was coupled CRISPR/Cas9 unit with the *OsC1^PLSr^* unit, and the high-level expression of Cas9 was a good indication of anthocyanin accumulation of edited plants [22]. Thus, if the purple leaf sheath *OsC1^PLSr^* was applied to rice breeding, the efficiency of rice hybrid breeding would be greatly improved, and the yield of hybrid rice would be increased. These results provided a basis for further understanding the genetic mechanism of leaf sheath color as well as its potential utilization in a two-line hybrid rice breeding system.

## 4. Materials and Methods

### 4.1. Plant Materials and Genetic Population Construction

A purple leaf sheath two-line male sterile line Hang93S (H93S) was used as the female parent, and two green leaf sheath conventional lines (R1173, YHSM) were used as male parental lines. H93S hybrid with R1173 and YHSM to produce the F_1_ generations, and two F_1_s self-pollinated to produce F2-1, and F2-2 populations, respectively. Both F2 populations, each of which consists of 1000 plants with no replication, were used to map the purple leaf sheath gene (*OsC1^PLSr^*). H93S and R1173 were used for leaf sheath histology analysis, anthocyanin content analysis, and UV-C irradiation treatment. All the plant materials were cultivated in the experimental field at South China Agricultural University (Guangzhou, China), followed by routine field management.

### 4.2. BSA Sequencing and Association Analysis

At the tillering stage, purple leaf sheath bulks (PLSBs) and green leaf sheath bulks (GLSBs) selected from the F2 population were constructed by pooling equivalent amounts of DNA from 50 purple and 50 green individuals. The healthy leaf sheaths of three parents (H93S, R1173, and YHSM) were collected to construct parental pools. The genomic DNA was extracted by CTAB for BSA sequencing. All DNA of two PLSBs, two GLSBs, and three parental pools were constructed into a library according to the manufacturer’s recommended protocol. BSA sequencing was performed using whole genome sequencing on HiSeq 2000 platform (Illumina) by Biomarker Technologies Co., Ltd. (Beijing, China). Since all the parents (H93S, YHSM, and R1173) are indica lines, R498 was therefore selected as the reference genome (http://mbkbase.org/R498/, accessed on 10 June 2021). Sequencing data were subjected to quality control and mapped to R498. The raw reads of the fast format were first processed through a series of quality control procedures to remove low-quality paired reads. Before association analysis, SNPs and InDels must be filtered. Firstly, SNPs (InDels) with multiple genotypes were screened; Secondly, SNPs (InDels) with read support of less than 4 were filtered out. Thirdly, mixing pool genotype SNPs (InDels) were screened from the recessive mixing pool genotype SNPs (InDels) of non-recessive parents. Finally, high-quality and credible SNPs and InDels were obtained for subsequent analysis. Using △All-index (△SNP-index and △Indel-index) and ED (Euclidean Distance), the region linked to the target gene was mapped by comprehensive analysis.

### 4.3. Screening of Candidate Genes

The association regions obtained from two F2 populations were comprehensively analyzed, and the intersection of the association regions located by both populations was selected as the candidate region of the purple leaf sheath gene. The expression profile of the public database CREP (http://crep.ncpgr.cn/, accessed on 5 December 2022) was used to screen candidate genes. The Rice Genome Annotation Project database (RGAP) annotates the putative functions of candidate genes. In order to confirm the final candidate genes, qRT-PCR analysis was used to verify their expression at the heading stage.

### 4.4. Histological, Anthocyanin Content, and Genetic Analysis

At the heading stage, longitudinal sections of rice leaf sheath were obtained by freehand sectioning, and the distribution of anthocyanins in leaf sheath cells was observed by using a digital microscope (OLYMPUS CX31). Anthocyanins were extracted from 0.5 g of finely ground leaf sheath tissue as described by Nakatskaa et al. [23]. Anthocyanin content (u.g^−1^) = OD/w, wherein “w” is the fresh weight of the leaf sheath. The leaf sheath color separation ratio in two F2 populations was investigated and calculated by the Chi-square test.

### 4.5. Sequence Analysis and Structure Analysis of OsC1^PLSr^

Specific primers linked to the *OsC1^PLSr^* gene were used to amplify gene sequences separately from the three parental lines (H93S, R1173, and YHSM) via using Phanta^®^ Max Super-Fidelity DNA Polymerase. Sequence alignment was performed with SnapGene. The CDS of *OsC1^PLSr^* from the purple and green leaf sheath parental lines were cloned and subjected to alignment by SnapGene. Structure analysis of the OsC1^PLSr^ protein was performed by using the SMART database (www.smart.embl-heidelberg.de, accessed on 5 December 2022).

### 4.6. CRISPR/Cas9 Generated the OsC1^PLSr^ Mutant Plants

Based on the conversed structure of OsC1^PLSr^ protein, an independent site in the exon 3 was designed as CRISPR/Cas9 spliced sites by CRISPR–P [2]. The fragment containing the target was recombined with the pRGEB32 vector, which was digested by *Bsa*I. Through the use of Agrobacterium tumefaciens, the constructs were transformed into H93S [24].

### 4.7. Transcription Activity Assay and Toxicity Detection in Yeast Report System

In order to determine the OsC1^PLSr^ transcriptional activity domain, various regions of the OsC1^PLSr^ protein coding sequence, including the R2-MYB domain (MYB1, 1-65aa), R3-MYB domain (MYB2, 66-144aa), C-terminal region (C,145-272aa) and the full-length OsC1^PLSr^ were amplified and inserted into *EcoRI/BamHI*-digested pGBKT7 vector. The recombination vectors and the negative control pGBKT7 (empty vector) were transformed into yeast strain AH109. The positive transformants were grown on SD/-Trp solid medium and SD/-Trp-His-Ade solid medium for three days at 30 °C. χ-α-gal was used to determine the transcription activation activity of the OsC1^PLSr^ protein regions in the yeast expression system. Besides, in order to determine whether the OsC1^PLSr^ protein produced a toxic effect on yeast growth, the yeast strains transformed with the recombination vectors pGBKT7-OsC1^PLSr^ (BD-OsC1^PLSr^) and others with the negative control pGBKT7 (empty vector, BD) were cultured with SD/-Trp liquid medium, respectively, which were shaken by shaker incubator (TENSUC). The OD_600_ values were measured at five-time points (5 h, 10 h, 15 h, 20 h, and 25 h), and the growth curve was drawn.

### 4.8. RNA Extraction and Quantitative qRT-PCR

Total RNA was extracted from the leaf sheath of H93S across the entire growth period; first, cDNA was synthesized by using the GoScript™ (Fisher Scientific, Waltham, MA, USA) Reverse Transcription System (Promega). The qRT-PCR verification was performed with three techniques repeated. The AceQ^®^ qPCR SYBR^®^ Green Master Mix was used for the reaction on a StepOnePlus^®^ Real-Time PCR System 272007300 (applied biosystems^®^ life technologies^TM^, Carlsbad, CA, USA). Differences in gene expression were calculated using the 2^−ΔΔCt^ method. The actin gene was used as an internal reference control. The expression level of eight structural genes (*ANS*, *DFR, F3H*, *F3′H*, *F3′5′H*, *PAL*, *CHS,* and *CHI*) and candidate genes of the leaf sheath of H93S were analyzed by qRT-PCR.

## 5. Conclusions

The purple leaf sheath is a quality trait that can be recognized by the naked eye at the seedling stage. The application of purple leaf sheaths in breeding is of great significance. In this study, two F2 populations from the crossing of purple leaf sheath with green leaf sheath were constructed, BSA sequencing was performed, and candidate region *qPLSr6* was located. *OsC1^PLSr^* was found to be a potential candidate gene. The constructed knockout mutant showed a purple sheath deletion phenotype, indicating that *OsC1^PLSr^* is closely related to the purple sheath phenotype. The results of the yeast report system showed that *OsC1^PLSr^* had a transcriptional activation function. The discovery of this result laid the foundation for revealing the molecular regulation mechanism of the purple leaf sheath.

## Figures and Tables

**Table 1:** Genetic analysis of the purple leaf sheath plants in two F2 segregating populations.

Population Name	Tested Plants	Purple Plants	Green Plants	Mendelian Expectations	χ^2^	χ^2^_(0.05, 1)_
F2-1 (H93S/R1173)	500	390	110	3:1	2.40	3.84
F2-2 (H93S/YHSM)	500	388	112	3:1	1.80	3.84

Note: χ^2^ <  χ^2^_(0.05, 1)_ is considered as significant.

**Table 2:** The data quantity and quality in BAS-seq.

Samples	Filtered Dates	Q30	GC	Mapped	Ave_Depth	Coverage
R1173	42,356,107	90.60%	44.45%	98.01%	28×	94.95%
F2-1 (P)	55,322,041	90.32%	44.40%	97.96%	37×	97.25%
F2-1 (G)	60,695,930	90.94%	44.77%	98.49%	40×	97.23%
H93S	36,790,415	91.30%	44.33%	98.56%	25×	94.85%
F2-2 (P)	55,367,274	91.27%	44.54%	98.58%	37×	97.33%
F2-2 (G)	56,536,080	91.08%	44.40%	98.50%	38×	96.76%
YHSM	43,800,417	91.33%	44.36%	98.56%	30×	95.09%

Note: P, purple leaf sheath plants in the F2 population; G, green leaf sheath plants in the F2 population; Q30, the bases of quality value greater than or equal to 30 in the total base number; GC, the GC content of the samples; Mapped, the percentage of clean reads mapped to the reference genome R498 in the total clean reads; Ave depth, average coverage depth of samples; coverage, the ratio of the bases above given depth to the total bases in the reference genome.

**Table 3:** Credible and high-quality SNPs/InDels in BSA-seq.

Population	FT	TL	MG	RSL4	CGMP	RPNP	CHQS/I
F2-1	SNPs	1,432,222	1961	21,971	443,822	288,956	675,512
	InDels	312,912	8875	10,217	97,239	56,207	140,374
F2-2	SNPs	1,251,158	1362	25,629	478,438	278,933	466,796
	InDels	274,708	6863	10,764	105,401	54,942	96,738

Note: FT, filtered type; TL, total loci; MG, multiple genotypes; RSL4, read support less than 4; CGMP, consistent genotypes among mixed pools; RPNP, recessive mixed pool genes not from recessive parents; CHQS/I, credible and high-quality SNPs/InDels.

**Table 4:** Statistics information of candidate association region in BSA-seq.

Population	CRAT	Chr.	Start Position	End Position	Size (Mb)	Genes
F2-1	SNP AA	6	3,330,000	8,720,000	5.39	1415
	InDel AA	6	3,730,000	8,550,000	4.82	1274
F2-2	SNP AA	6	4,030,000	9,060,000	5.03	1275
	InDel AA	6	4,610,000	6,030,000	1.42	415

Note: CRAT, candidate region analysis type; SNP AA, SNP association analysis; InDel AA, InDel association analysis.

**Table 5:** Gene annotation of the 27 candidate genes by using Rice Genome Annotation Project.

Genes	MSU_Locus	Annotation
*OsR498G0611776200.01*	*LOC_Os06g09540*	SAC domain containing protein, putative, expressed
*OsR498G0611780600.01*	*LOC_Os06g09620*	expressed protein
*OsR498G0611782900.01*	*LOC_Os06g09679*	chaperonin, putative, expressed
*OsR498G0611789000.01*	*LOC_Os06g09850*	25.3 kDa vesicle transport protein, putative, expressed
*OsR498G0611790200.01*	*LOC_Os06g09890*	smr domain containing protein, expressed
*OsR498G0611790800.01*	*LOC_Os06g09900*	expressed protein
*OsR498G0611792500.01*	*LOC_Os06g09930*	G protein coupled receptor, putative, expressed
*OsR498G0611812900.01*	*LOC_Os06g10340*	autophagy-related protein 12, putative, expressed
*OsR498G0611814100.01*	*LOC_Os06g10350*	MYB family transcription factor, putative, expressed
*OsR498G0611822700.01*	*LOC_Os06g10520*	pantothenate kinase, putative, expressed
*OsR498G0611830400.01*	*LOC_Os06g10620*	transcription elongation factor SPT5 homolog 1, putative, expressed
*OsR498G0611870100.01*	*LOC_Os06g11620*	RNA recognition motif containing protein, putative, expressed
*OsR498G0611870800.01*	*LOC_Os06g11640*	serine/threonine-protein phosphatase 2A activator 2, putative, expressed
*OsR498G0611835200.01*	*LOC_Os06g10690*	PHD-finger domain containing protein, putative, expressed
*OsR498G0611836500.01*	*LOC_Os06g10750*	integral membrane protein DUF6 containing protein, expressed
*OsR498G0611837500.01*	*LOC_Os06g10790*	lectin-like receptor kinase, putative, expressed
*OsR498G0611842100.01*	*LOC_Os06g10900*	DELLA protein RGL3, putative, expressed
*OsR498G0611842900.01*	*LOC_Os06g10910*	xyloglucan fucosyltransferase, putative, expressed
*OsR498G0611843200.01*	*LOC_Os06g10920*	xyloglucan fucosyltransferase, putative, expressed
*OsR498G0611848000.01*	*LOC_Os06g11020*	tic22-like family domain containing protein, expressed
*OsR498G0611849000.01*	*LOC_Os06g11040*	expressed protein
*OsR498G0611856800.01*	*LOC_Os06g11270*	Anthocyanidin 3-O-glucosyltransferase, putative, expressed
*OsR498G0611859800.01*	*LOC_Os06g11330*	OsMADS55—MADS-box family gene with MIKCc type-box, expressed
*OsR498G0611860600.01*	*LOC_Os06g11370*	mediator of RNA polymerase II transcription subunit 6, putative, expressed
*OsR498G0611862900.01*	*LOC_Os06g11410*	cyclin, putative, expressed
*OsR498G0611871200.01*	*LOC_Os06g11660*	phosphate-induced protein 1 conserved region domain containing protein, expressed
*OsR498G0611864700.01*	*LOC_Os06g11430*	GRAM domain containing protein, expressed

## Data Availability

The data presented in this study are available upon request from the corresponding authors.
